# Apical-basal polarity of the spectrin cytoskeleton in the *C. elegans* vulva

**DOI:** 10.17912/micropub.biology.000863

**Published:** 2023-06-14

**Authors:** Trevor J. Barker, Fung-Yi Chan, Ana X. Carvalho, Meera V. Sundaram

**Affiliations:** 1 Department of Genetics, University of Pennsylvania, Philadelphia, Pennsylvania, United States; 2 i3S-Instituto de Investigação e Inovação em Saúde, University of Porto, Porto, Portugal; 3 IBMC-Instituto de Biologia Molecular e Celular, University of Porto, Porto, Portugal

## Abstract

The *C. elegans* vulva is a polarized epithelial tube that has been studied extensively as a model for cell-cell signaling, cell fate specification, and tubulogenesis. Here we used endogenous fusions to show that the spectrin cytoskeleton is polarized in this organ, with conventional beta-spectrin ( UNC-70 ) found only at basolateral membranes and beta heavy spectrin ( SMA-1 ) found only at apical membranes. The sole alpha-spectrin ( SPC-1 ) is present at both locations but requires SMA-1 for its apical localization. Thus, beta spectrins are excellent markers for vulva cell membranes and polarity.

**Figure 1. Apical-basal polarity of the spectrin cytoskeleton in the C. elegans vulva:**
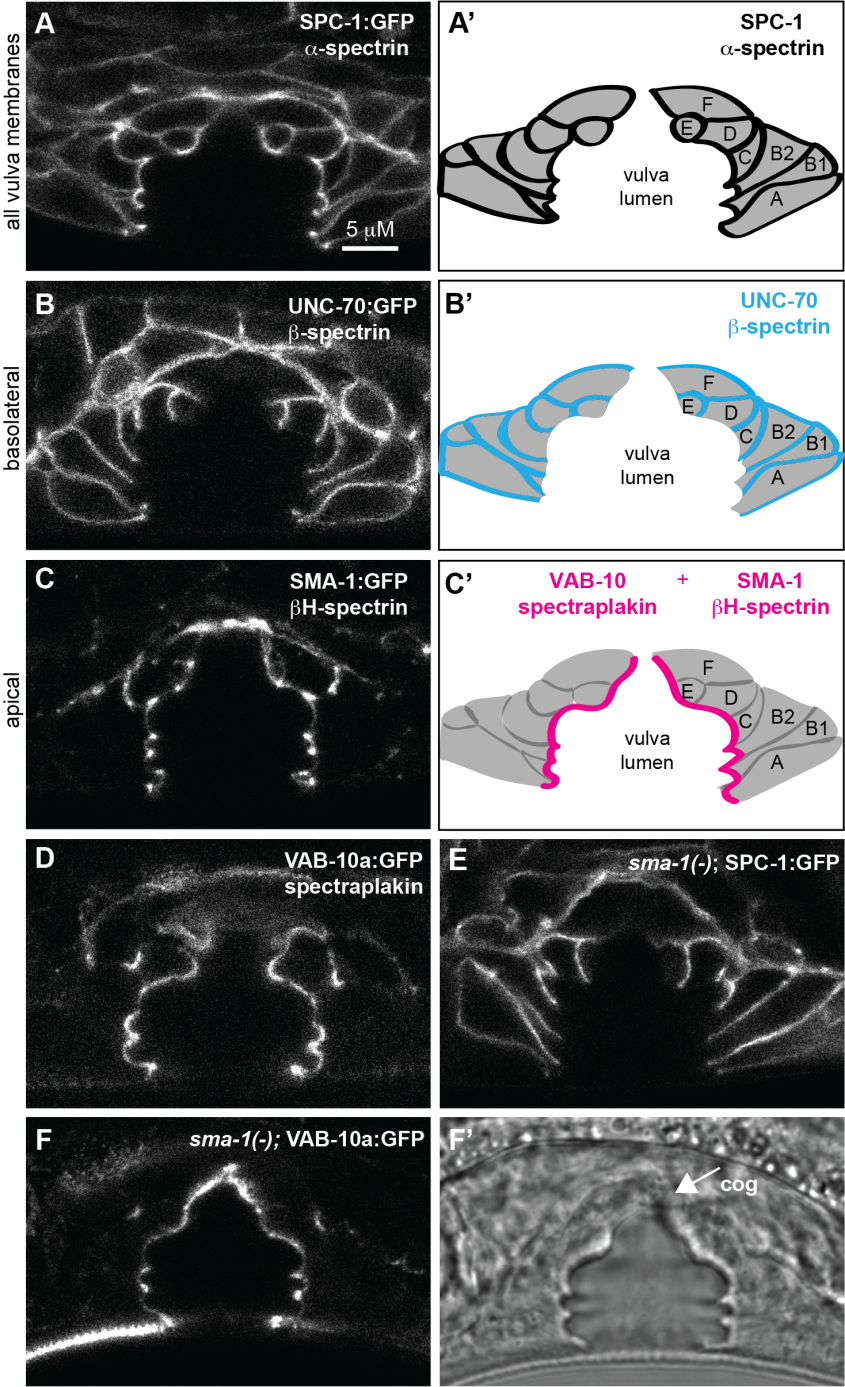
A, A’) SPC-1 /α-spectrin marks all vulva cell membranes; A’ is a cartoon tracing of the 7 vulA-vulF rings. B, B') UNC-70 /β-spectrin marks basolateral membranes. C, C') SMA-1 /βH-spectrin marks apical cell membranes. B' and C' cartoons are drawn in comparison to A’. D) VAB-10a/spectraplakin marks apical vulva cell membranes. E, F) SMA-1 /βH-spectrin is required for apical localization of SPC-1 /α-spectrin (E) but not VAB-10a/spectraplakin (F). F' shows DIC channel, with disorganized tissue occluding the connection between the gonad and the dorsal apex of the vulva (cog phenotype, arrow). All images are single confocal Z-slices through the medial portion of the mid-L4 vulva tube and are representative of at least n=10 animals examined per genotype.

## Description

The spectrin cytoskeleton lines the cytoplasmic sides of cell plasma membranes (Teliska and Rasband 2021; Lorenzo *et al.* 2023). There, spectrins serve as linkers to connect plasma membranes and transmembrane proteins to the actin cytoskeleton (Xu *et al.* 2013; Machnicka *et al.* 2014; Liem 2016; Li *et al.* 2023). They play numerous roles in membrane and cytoskeletal organization and stability, tissue mechanics, and morphogenesis (Hammarlund *et al.* 2007; Bennett and Healy 2008; Duan *et al.* 2018; Mylvaganam *et al.* 2020, 2022; Krueger *et al.* 2020)

Spectrins exist as tetramers composed of two dimers, each with an alpha (α) and beta (β) subunit (Liem 2016; Lorenzo *et al.* 2023). Vertebrates have multiple α-spectrin and β-spectrin genes. Invertebrates such as *Drosophila melanogaster* and *Caenorhabditis elegans* have just one α-spectrin and two β-spectrin genes, a conventional β-spectrin and a larger or “heavy” β-spectrin (βH). *C. elegans* α-spectrin is encoded by *spc-1* , and β-spectrin and βH-spectrin are encoded by *unc-70* and *sma-1* , respectively (McKeown *et al.* 1998; Hammarlund *et al.* 2000; Norman and Moerman 2002). SPC-1 functions with either UNC-70 or SMA-1 to control different processes. In general, UNC-70 is widely expressed and plays key roles in neuron and muscle structure (Hammarlund *et al.* 2000, 2007; Moorthy *et al.* 2000; Jia *et al.* 2019), while SMA-1 is expressed more specifically in epithelial cells and affects tissue morphogenesis (Praitis *et al.* 2005). We also discovered a role for SMA-1 in blastomere cytokinesis (Sobral *et al.* 2021; Silva *et al.* 2023).

In *D. melanogaster* , the two β-spectrins exhibit apical-basal polarity in epithelial cells, with conventional β-spectrin found primarily at basolateral membranes and βH-spectrin found primarily at apical membranes, while α-spectrin is found at both locations (Thomas and Kiehart 1994; Thomas and Williams 1999) . *D. melanogaster* βH-spectrin can also influence apical localization of other factors (Zarnescu and Thomas 1999; Dubreuil *et al.* 2000; Pogodalla *et al.* 2021). In *C. elegans* , immunolocalization and/or transgenic reporter studies in the embryo detected UNC-70 /β-spectrin at many sites of cell-cell contact but SMA-1 /βH-spectrin only at apical membranes (Moorthy *et al.* 2000; Norman and Moerman 2002; Praitis *et al.* 2005), suggesting that apical vs. basal partitioning of different β-spectrins is conserved; however, there has been limited analysis of spectrin polarity in larval or adult epithelia (Wirshing and Cram 2018) .

Here we visualized the spectrin cytoskeleton in the *C. elegans* vulva. The vulva is a polarized epithelial tube used for egg-laying. It has been studied extensively as a model for cell-cell signaling, cell fate specification, and tubulogenesis (Sharma-Kishore *et al.* 1999; Schindler and Sherwood 2013; Gauthier and Rocheleau 2017). Vulva anatomy is well characterized and apical vs. basal cell surfaces can be distinguished easily using simple light microscopy of live animals.

Endogenous GFP fusions for SPC-1 /α-spectrin and UNC-70 /β-spectrin have been reported previously (Jia *et al.* 2019). We used CRISPR/Cas9 to tag the endogenous SMA-1 /βH-spectrin protein with GFP (Methods) and then examined the localization patterns of all three fusions in the developing vulva. At the mid-L4 larval stage, vulva cells are organized into a series of 7 stacked rings (named vulA-vulF), surrounding a central lumen cavity (Sharma-Kishore *et al.* 1999). SPC-1 ::GFP outlined all 7 rings ( Fig. 1A, A ’) and was present near apical (luminal) membranes and also at sites of cell-cell contact (basolateral membranes). In contrast, UNC-70 ::GFP was present only at basolateral membranes ( Fig. 1B, B '), while SMA-1 ::GFP was present only at apical membranes ( Fig. 1C, C '). We showed previously that the spectrin-related protein VAB-10a/spectraplakin also localizes apically in the vulva (Cohen *et al.* 2020) ( Fig. 1D ). Thus, β-spectrin and βH-spectrin localize in a polarized fashion in the vulva, and the βH-spectrin and spectraplakin patterns appear very similar ( Fig. 1C ').

In agreement with SMA-1 / SPC-1 tetramer formation, we found that SMA-1 /βH-spectrin is needed to localize SPC-1 /α-spectrin to apical membranes. In all *sma-1 ( ru18 )* null mutants examined, SPC-1 ::GFP failed to localize to apical membranes and was found only at basolateral membranes ( Fig. 1E ), resembling the pattern seen with UNC-70 ::GFP ( Fig. 1B ). In contrast, VAB-10a::GFP remained unchanged compared to wild type ( Fig. 1D,F ). Therefore, SMA-1 is not required for overall polarity of the vulva.

The similarly polarized distributions of β-spectrin and βH-spectrin in *C. elegans* and *D. melanogaster* suggest this may be an ancestral feature of metazoan β-spectrins that has been conserved across the many hundreds of millions of years since the evolutionary divergence of nematodes and insects. Some vertebrate tissues also exhibit a polarized spectrin cytoskeleton, but the clear distinction between conventional β-spectrins and βH spectrin appears to have been lost in vertebrates, since either can be found apically or basolaterally (Stabach and Morrow 2000; Cortese *et al.* 2017; Mylvaganam *et al.* 2020, 2022).

The roles of polarized spectrins in vulva development remain to be determined. Vulva cell types must divide in a stereotyped fashion, migrate inward to form a luminal cavity, and then assemble into doughnut-like rings via cell-cell fusion (Sulston and Horvitz 1977; Sharma-Kishore *et al.* 1999). These rings adopt specific shapes and undergo stereotypical movements during morphogenesis. The vulva also must connect appropriately with the uterus to allow the passage of eggs, and with the sex muscles and neurons that control egg-laying (Trent *et al.* 1983; Schindler and Sherwood 2013). Many of these cell behaviors depend on the actin cytoskeleton and/or on interactions with apical or basal extracellular matrices (Bulik and Robbins 2002; Farooqui *et al.* 2012; Hagedorn *et al.* 2013; Morrissey *et al.* 2014; Yang *et al.* 2017; Cohen *et al.* 2020). We observed a variety of vulva shape abnormalities in *sma-1* mutants (e.g. Fig. 1F '), which may involve some of the above processes and will be described in a separate report. In the meantime, the spectrin fusions described here will be useful membrane markers for studies of vulva development.

## Methods

*Caenorhabditis elegans* strains were grown at 20˚C under standard conditions (Brenner 1974) . For immobilization during imaging, L4 larvae were mounted on 2% agar pads containing 20 mM sodium azide, along with 10 mM levamisole in a drop of M9 buffer. Confocal z-stacks were collected with a 63X Plan Apo objective (HC PL APO CS2 63x/1.30 GLYC) on a Leica TCS SP8 confocal microscope. Images were processed in FIJI (Schindelin *et al.* 2012) and the panels assembled with Adobe Illustrator.

CRISPR/Cas9-mediated genome editing to tag endogenous SMA-1 with GFP was performed by Suny Biotech. The tag is inserted at the SMA-1 C-terminus, immediately preceding the stop codon, as shown below. This endogenous fusion is functional based on normal body morphology, brood size and embryonic viability of the homozygotes.

1) Wild type sequence:

TTATTCAAGCGTGGATCCAAACATTCAAAG* **TAG** atacctcaccacacgctgatcttcata

Bold TAG is the stop codon of *sma-1* ; asterisk is where the GFP was inserted

2) Precise sequence knock-in, *sma-1 ( syb4954 [ SMA-1 ::GFP])* V

***C*** T ***G*** TTCAAGCGTGGATCCAAACATTCAAAG AGTAAAGGAGAAGAACTTTTCACTGGAGTTGTCCCAATTCTTGTTGAATTAGATGGTGATGTTAATGGGCACAAATTTTCTGTCAGTGGAGAGGGTGAAGGTGATGCAACATACGGAAAACTTACCCTTAAATTTATTTGCACTACTGGAAAACTACCTGTTCCATGGgtaagtttaaacatatatatactaactaaccctgattatttaaattttcagCCAACACTTGTCACTACTTTCTgTTATGGTGTTCAATGCTTcTCgAGATACCCAGATCATATGAAACgGCATGACTTTTTCAAGAGTGCCATGCCCGAAGGTTATGTACAGGAAAGAACTATATTTTTCAAAGATGACGGGAACTACAAGACACgtaagtttaaacagttcggtactaactaaccatacatatttaaattttcagGTGCTGAAGTCAAGTTTGAAGGTGATACCCTTGTTAATAGAATCGAGTTAAAAGGTATTGATTTTAAAGAAGATGGAAACATTCTTGGACACAAATTGGAATACAACTATAACTCACACAATGTATACATCATGGCAGACAAACAAAAGAATGGAATCAAAGTTgtaagtttaaacatgattttactaactaactaatctgatttaaattttcagAACTTCAAAATTAGACACAACATTGAAGATGGAAGCGTTCAACTAGCAGACCATTATCAACAAAATACTCCAATTGGCGATGGCCCTGTCCTTTTACCAGACAACCATTACCTGTCCACACAATCTGCCCTTTCGAAAGATCCCAACGAAAAGAGAGACCACATGGTCCTTCTTGAGTTTGTAACAGCTGCTGGGATTACACATGGCATGGATGAACTATACAAA **TAG** atacctcaccacacgctgatcttcata

Bold italics indicate silent mutations; underline indicates the GFP sequence.

## Reagents

Strains used:

GOU2043 *vab-10 ( cas602 [VAB-10a::GFP])* I

GOU2936 *spc-1 ( cas815 [ SPC-1 ::GFP])* X

GOU3103 *unc-70 ( cas962 [ UNC-70 ::GFP)]* V

PHX4954 *sma-1 ( syb4954 [ SMA-1 ::GFP])* V

UP4241 *sma-1 ( ru18 ) V; spc-1 ( cas815 [ SPC-1 ::GFP])* X

UP4252 *vab-10 ( cas602 [VAB-10a::GFP]) I; sma-1 ( ru18 )* V
